# Improved adherence and treatment outcomes with an engaging, personalized digital therapeutic in amblyopia

**DOI:** 10.1038/s41598-020-65234-3

**Published:** 2020-05-20

**Authors:** Scott Xiao, Eric D. Gaier, Malcolm L. Mazow, Ann U. Stout, Dean A. Travers, Endri Angjeli, Hank C. Wu, Gil Binenbaum, David G. Hunter

**Affiliations:** 1 Luminopia, Inc., Cambridge, MA 02139 USA; 2 Massachusetts Institute of Technology, Department of Brain and Cognitive Sciences, Picower Institute for Learning and Memory, Cambridge, MA 02139 USA; 3 Houston Eye Associates, Houston, TX 77025 USA; 4 Children’s Hospital of Philadelphia, Division of Ophthalmology, Philadelphia, PA 19104 USA

**Keywords:** Visual system, Eye diseases, Biomedical engineering

## Abstract

Given the prevalence of poor adherence to therapy and the biases of self-reporting across healthcare, we hypothesized that an engaging, personalized therapy may improve adherence and treatment outcomes in the home. We tested this hypothesis in the initial indication of amblyopia, a neurodevelopmental disorder for which available treatments are limited by low adherence. We designed a novel digital therapeutic that modifies patient-selected cinematic content in real-time into therapeutic visual input, while objectively monitoring adherence. The therapeutic design integrated a custom-designed headset that delivers precise visual input to each eye, computational algorithms that apply real-time therapeutic modifications to source content, a cloud-based content management system that enables treatment in the home, and a broad library of licensed content. In a proof-of-concept human study on the therapeutic, we found that amblyopic eye vision improved significantly after 12 weeks of treatment, with higher adherence than that of available treatments. These initial results support the utility of personalized therapy in amblyopia and may have broader relevance for improving treatment outcomes in additional indications.

## Introduction

Given the prevalence of poor adherence to therapy across healthcare^1^, novel approaches to increase patient engagement with therapy may improve treatment outcomes in a multitude of conditions. In recent years, personalized medicine has largely focused on increasing therapeutic efficacy^2,3^. However, since successful treatment outcomes, particularly in the home, require high adherence in addition to efficacy, personalizing therapy to increase patient engagement may be as impactful. The development of a novel and engaging digital therapeutic that supports personalization of therapy in the home could be a scalable platform to test this hypothesis. Here we report on the design and development of such a digital therapeutic for the initial indication of amblyopia and present results from a proof-of-concept human study evaluating its safety and efficacy.

Amblyopia, known colloquially as “lazy eye”, is a neurodevelopmental disorder of the visual system resulting from abnormal visual experience during development. Amblyopia is characterized by reduced vision in one eye or both eyes that cannot be explained by structural ocular or optic nerve abnormalities alone. It is the most prevalent cause of reduced vision in one eye (monocular visual acuity) in children and young adults, affecting 1–5% of the population^4^. In addition to having reduced visual acuity, patients with amblyopia may also suffer from deficiencies in focusing (accommodation), fixation stability, binocularity, reading fluency, depth perception (stereoacuity), and contrast sensitivity^5–8^. From a health economics standpoint, amblyopia costs the United States $7.4 billion per year in lost earning potential^9^.

The current standard of care treatment for amblyopia beyond refractive correction (optical treatment) targets the stronger (fellow) eye, through patching or cycloplegic (blurring) drops such as atropine, to promote usage of the weaker (amblyopic) eye^4^. These approaches, despite a long history of use, are limited by high rates of failure and relapse. In younger children aged 3 to 7 years treated with patching, 54% suffered from residual amblyopia at age 10^10^ and 40% at age 15^11^. In older children aged 7 to 12 years treated with patching, 74% had residual amblyopia at the time of treatment termination^12^. In teenagers aged 13 to 17 years, patching is only minimally effective^13^.

One recognized explanation for the limitations of these treatments is poor adherence. When adherence is patient-reported, only half of patients complete more than 75% of prescribed patching^12^. When objectively monitored, adherence is even lower, averaging 44%, with patients skipping 42% of the days they were prescribed patching^14^. Adherence to prescribed treatment is essential because amblyopia treatment is highly dose-dependent. Patching, for example, has a linear dose-response curve of 1 logMAR line gain in visual acuity per 120 hours of patching^15^. Adherence to amblyopia treatment is also challenging due to the pediatric patient population and because treatment is largely received in the home. Nevertheless, some patients who are highly adherent to patching still fail to improve^15^, suggesting underlying deficiencies in the mechanism of action of patching as well.

More recently, dichoptic treatments, which rebalance the visual input between the eyes in an attempt to promote binocularity, have shown promise in several pilot studies as a potentially more effective alternative to patching^16–21^. The theoretical underpinnings for these approaches are based on the observation, first reported by Baker *et al*.^16^, that patients with amblyopia have latent binocularity and tend to function monocularly due to interocular suppression^17^. It has been hypothesized that by balancing visual stimuli to account for interocular suppression, binocularity, and subsequently amblyopic eye visual acuity, can be improved^18^.

Dichoptic treatments require presentation of targeted visual input to each eye, providing an opportunity for engaging treatment delivery mechanisms. However, some studies on dichoptic treatments delivered through video games^22^, but see also^19,21^, have also been hindered by poor adherence, possibly because patients tire of playing the same game repeatedly. Subsequent studies, first by Li *et al*.^23^ and Sauvan *et al*.^24^, have tested dichoptic treatments delivered through cinematic content^23,25,26^ instead of video games as an alternative approach to improve adherence. However, these treatments have relied on a limited library of publicly accessible content, which may have hindered engagement.

We hypothesized that a novel digital therapeutic that delivers dichoptic treatment through patient-selected cinematic content in a head-mounted display would improve both adherence and treatment outcomes in the home for children with amblyopia. As such, we designed and developed a device – the “Luminopia One” therapeutic – that incorporates the following critical components. We designed a custom headset tailored for the pediatric population that includes a pair of viewing lenses for dichoptic presentation and a smartphone for displaying visual input. Computational algorithms on the smartphone modify patient-selected content in real-time into therapeutic visual input, and the input is delivered precisely to each eye through the headset. An on-demand, cloud-based content management system enables treatment in the home, and a broad library of 700+ hours of cinematic content licensed from leading media companies allowed patients a substantial degree of choice. Usage of the therapeutic was automatically captured, allowing for objective adherence reporting, and a clinical dashboard provided healthcare professionals with real-time adherence data to follow-up with patients as needed. As far as we are aware, this is the first attempt to combine a head mounted display with a broad library of cloud-based video content to allow for personalized amblyopia therapy at home for an extended period of time.

We also report the results of a proof-of-concept human study on the Luminopia One therapeutic. The primary objective of this study was to assess the efficacy of the therapeutic to improve amblyopic eye best-corrected visual acuity (BCVA) of patients aged 4 to 7. Secondary objectives included assessment of patient adherence and patient satisfaction with the therapeutic. Safety measures included monitoring for new double vision (diplopia), new or worsening eye misalignment (heterotropia), worsening visual acuity, or other unanticipated adverse events.

## Results – Therapeutic Design

### Head-mounted display

A head-mounted display was selected as the digital platform that met the requirements for precision and controls. Specifically, immersion in a head-mounted display allows for dichoptic presentation of visual input, in which different channels of computationally processed content can be presented to the fellow eye and the amblyopic eye. Additionally, given that amblyopia treatment is largely received in the home, the head-mounted display provides a self-contained, self-sufficient, and portable form factor for treatment.

**Figure 1:**
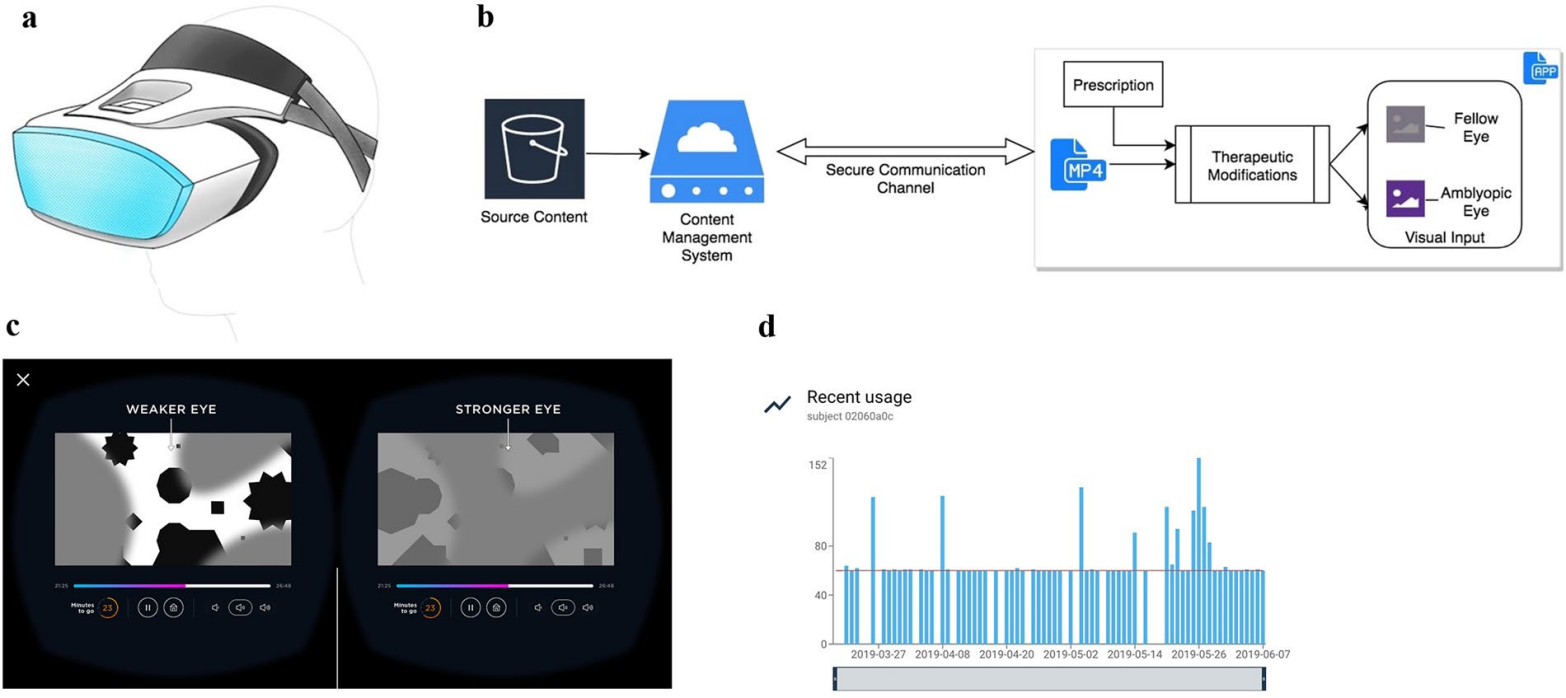
The Luminopia One Therapeutic. (**a**) Concept schematic of the custom headset. (**b**) Software architecture diagram for the therapeutic. (**c)** Schematic of visual input presented to each eye after therapeutic modifications have been applied to content input. (**d**) Modified screenshot displaying an example of a patient’s daily usage (in minutes) on the clinical dashboard.

Due to the absence of commercially available headsets designed for young children, we designed and manufactured a custom headset specifically for the target population of children aged 4 to 7 (Fig. 1a). The custom headset consisted of several standard components, including a viewing lens for each eye, a strap system for patients to secure the headset to their head, and a display unit to present visual input. Importantly, these components were designed to accommodate the unique physical and developmental characteristics of the pediatric population while also fitting over glasses.

Two critical design considerations were the ergonomics and optics of the headset. A unique halo-like design for the strap system shifted the vector of weight on the head from forwards to downwards. To minimize the disparity between the inter-lens distance of the headset and the interpupillary distance (IPD) of pediatric patients (significant disparity between the two can lead to image distortion and an induced prismatic effect), we designed a lens system with two settings: one for patients with smaller IPDs and one for patients with larger IPDs.

A commercially available LG G5 smartphone (LG Electronics Inc., Seoul, South Korea) was used as the display unit for the platform, and the custom headset contained a slot to hold the phone. A software application pre-installed onto the smartphone ran computational algorithms that modified source content into therapeutic visual input delivered to each eye through the headset. The software application required an internet connection enabled by the smartphone’s Wi-Fi connectivity.

### Computational algorithms

Computational algorithms were designed to apply therapeutic modifications in real-time to patient-selected cloud-based source content. The video player was designed to play videos encoded at 30 frames per second (fps) with a maximum time allowance of 16 milliseconds (ms) between renders. The time allowance for the video player was more stringent than that of standard web-based video players, since content in the therapeutic was rendered in a fully immersive, 360-degree environment with a 60 fps refresh rate.

When a video was selected by a patient, computational algorithms split the source content into two streams, one for each eye, and applied therapeutic modifications to each stream in real-time (Fig. 1b). To fulfill the 16 ms time allowance requirement, the computationally intensive therapeutic modifications were processed on the graphical processing unit (GPU) instead of the central processing unit (CPU). To ensure the video and audio streams remained in sync during playback, the video player was engineered to route audio directly from the source content to the hardware speakers, bypassing the high-latency Android layer. To our knowledge, this is the first implementation of cloud-based content being modified and rendered in real-time in a head-mounted display for therapeutic purposes.

Therapeutic modifications were applied in the same manner to all selected videos based on the patient’s prescription, which indicated which eye was amblyopic (Fig. 1c). Firstly, the RGB channels of each pixel were modified to reduce the contrast of the fellow eye input to 15% of the contrast of the amblyopic eye input. Secondly, dichoptic masks were superimposed onto the inputs for both eyes, such that parts of the video were only visible to the amblyopic eye, parts were only visible to the fellow eye, and parts were visible to both eyes. The masks were colored grey (RGB: 127, 127, 127) and had feathered edges. The therapeutic cycled through 6 unique pairs of dichoptic masks, with each pair applied for 30 seconds at a time. The treatment protocol, including the interocular relative contrast level, was set at the same level for all patients and remained constant throughout the study.

### Content management system

The content management system was designed to enable treatment in the home by supporting an array of source content formats, while maximizing image quality and security during playback. Firstly, the infrastructure consisted of a pre-processing pipeline that transcoded raw, high fidelity videos to the target video and audio codecs of the video player. These codecs were selected to provide high playback quality given the virtual environment in which videos were ultimately rendered. Transcoded videos were stored remotely in the cloud prior to patient selection, which allowed for a substantially larger library of content than otherwise feasible. When a video was selected by a patient in the headset, the infrastructure provided the video player with a signed and secured URL from which the video was streamed, and computational algorithms applied real-time modifications to the video. This design ensured that the source content remained secure and that videos could be streamed directly and remotely from the cloud.

### Licensed content

The content management system provided patients with access to a broad library of licensed content to choose from throughout the study. The content included 700+ hours of popular TV shows, movies, cartoons, and videos licensed from Public Broadcasting Service (PBS) Distribution, Sesame Workshop, DreamWorks Animation, A&E Network, Millimages, National Broadcasting Company (NBC), and Corus, and publicly accessible content on YouTube. All videos were categorized into age groups according to Official Parental Guidelines or Common Sense Media guidelines, and patients were only able to select videos labeled appropriate for their age.

### Clinical dashboard

A web-based clinical dashboard allowed various healthcare professionals to monitor each patient’s treatment throughout the study (Fig. 1d). The dashboard provided data on each patient’s daily usage in real-time and highlighted patients that were below a certain threshold of adherence in the past week.

## Results – proof-of-concept study

### Study design

The proof-of-concept human study focused on assessing the safety and efficacy of the Luminopia One therapeutic. All patients (*n* = 10, Table 1, Supplementary Data File S1) were prescribed the therapeutic to be used at home for 1 hour per day, 7 days per week, for 12 consecutive weeks. Patients returned for follow-up visits after 2, 4, 8, and 12 weeks of therapy. Amblyopic eye best-corrected visual acuity (BCVA) was tested at each follow-up visit. Adherence to the therapeutic was recorded automatically by the therapeutic and patient satisfaction was assessed using a standard questionnaire at the 12-week visit. The detailed protocol is further described in the Methods section.

**Table 1:** Patient characteristics.

Patient	Age	Sex	Refractive error (OD)	Refractive error (OS)	Age at diagnosis (years)	Total Prior Duration in Refractive Correction (months)	Prior Treatment Methods*	Prior Treatment Durations (months)*	Amblyopic Eye
**01**	4	F	+3.00 + 1.50 × 90	+ 3.50 + 1.50 × 90	4	4 months	Patching	1 month	OS
**02**	5	F	+5.00 + 2.00 × 89	+ 4.50 + 1.75 × 92	4	18 months	Patching, atropine	17 months	OD
**03**	5	F	+4.00 + 1.75 × 74	Plano	N/A**	7 months	Patching	6 months	OD
**04**	4	F	+1.50 + 1.50 × 60	Plano	2	23 months	Patching	21 months	OD
**05**	4	M	N/A**	N/A**	4	3 months	Patching	2 months	OS
**06**	6	M	+3.00 + 3.00 × 94	+ 1.00 + 0.50 × 105	5	11 months	Patching	11 months	OD
**07**	6	F	+4.25 + 0.25 × 92	+ 4.75 + 1.00 × 59	2	40 months	Patching, atropine	36 months	OS
**08**	7	M	+5.25 + 0.25 × 95	+ 4.75 + 0.50 × 86	6	54 months	Patching	4 months	OD
**09**	7	M	+3.00 + 2.25 × 51	+ 3.25 + 2.50 × 85	5	30 months	None	None	OS
**10**	6	F	− 4.25 + 4.50 × 105	+ 0.75	6	10 months	Patching	8 months	OD

*Prior treatment excludes optical treatment.

**Missing data.

### Visual acuity

Best-corrected visual acuity (BCVA) was assessed at each visit using an electronic visual acuity (EVA) system with standard research acuity tests and pre-programmed testing protocols. Visual acuity was recorded in logMAR and Snellen equivalents. Patients aged 4 to 6 were assessed using the ATS-HOTV protocol^27^ and patients aged 7 were assessed using the e-ETDRS protocol^28^. Both the ATS-HOTV and the e-ETDRS protocols have demonstrated high testability and test-retest reliability^27,28^. Mean (SD) amblyopic eye BCVA at baseline was 0.42 (0.12) logMAR (approx. Snellen 20/50 equivalent). All patients had undergone a period of optical treatment prior to study enrollment ≥3 months, and the median duration of prior refractive correction wear was 14.5 months. All but 1 patient had a prior history of amblyopia treatment consisting of patching and/or atropine penalization (Table 1).

**Figure 2:**
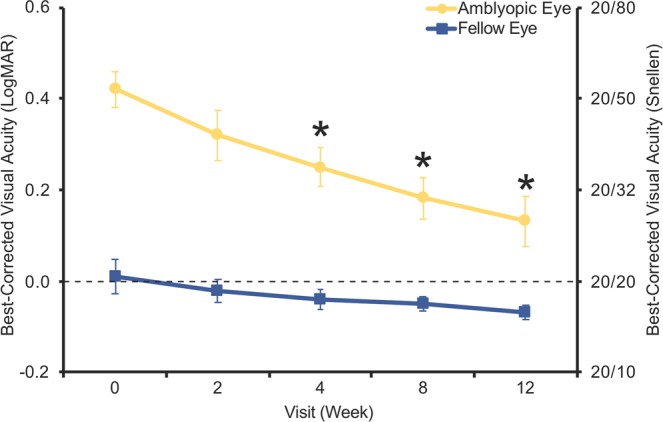
Visual acuity measurements. Mean best-corrected visual acuity in amblyopic and fellow eyes at each study visit across all 10 patients. Data are means ± SEM (*n* = 10). *p < 0.05 compared to Week 0 (baseline visit), Wilcoxon signed rank test for amblyopic eyes.

Amblyopic eye BCVA improved by 0.29 logMAR (2.9 logMAR lines, *p* < 0.01) from baseline to the 12-week visit (Fig. 2, Table 2); this equates to nearly three lines of improvement on the standard eye chart. In addition, 6 of 10 patients had resolution of their amblyopia such that final interocular difference in visual acuity was <0.3 logMAR.

**Table 2:** Patient measurements.

Patient	Ocular Alignment (prism diopters)	Stereoacuity (arcsec)	Amblyopic Eye BCVA baseline (logMAR)	Fellow Eye BCVA baseline (logMAR)	Amblyopic Eye BCVA final (logMAR)	Fellow Eye BCVA final (logMAR)
**01**	2 ET	3000	0.5	0.3	0.0	−0.1
**02**	Ortho	40	0.3	0.0	0.3	0.0
**03**	1 ET, 2 R/LHT*	3000	0.6	−0.1	0.3	−0.1
**04**	2 ET	10000	0.6	−0.1	0.4	−0.1
**05**	Ortho	3000	0.5	0.0	0.2	0.0
**06**	8 ET	40	0.3	0.1	0.0	0.0
**07**	Ortho	100	0.3	0.0	0.2	−0.1
**08**	Ortho	3000	0.3	0.0	0.1	−0.1
**09**	Ortho	3000	0.4	0.0	−0.1	−0.1
**10**	Ortho	40	0.4	−0.1	−0.1	−0.1

*Missing data.

Abbreviations: BCVA – best-corrected visual acuity, ET – esotropia, RHT – right hypertropia, LHT – left hypertropia.

### Adherence

**Figure 3:**
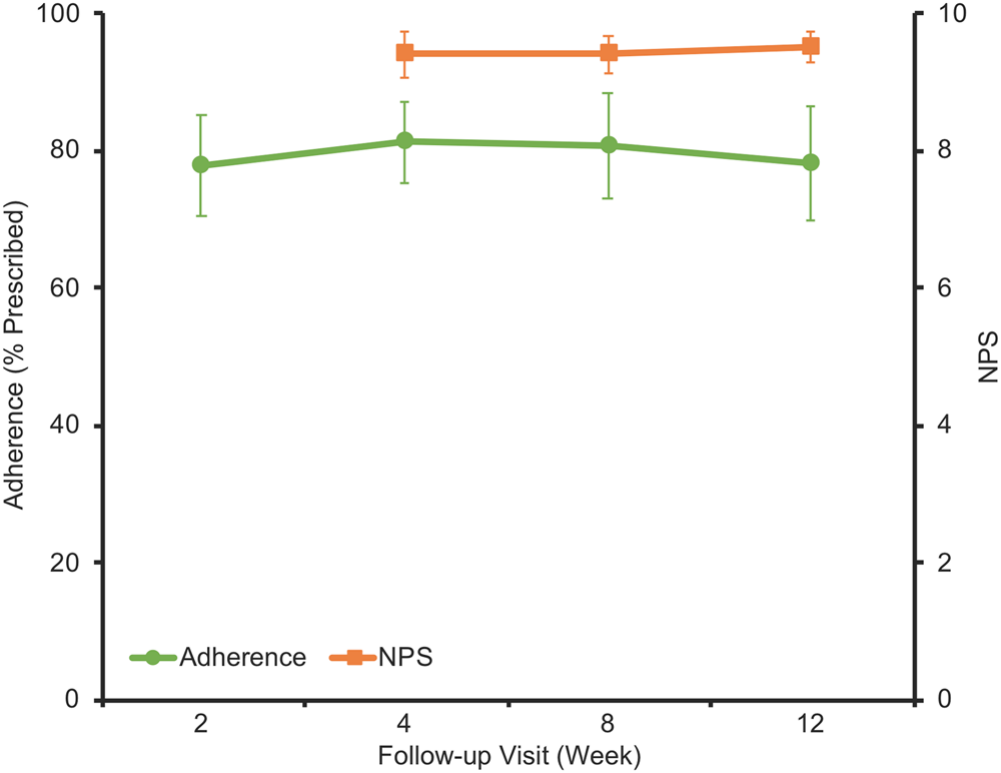
Adherence and patient satisfaction measurements. Mean adherence as a percentage of prescribed amount from Week 0 and mean response to NPS question at each follow-up visit across all patients. Data are means ± SEM (*n* = 10).

Daily usage for each patient was automatically recorded by the therapeutic throughout the study to the nearest minute. Adherence was calculated as a percentage of the total time prescribed (1 hour per day, 7 days per week for study duration) that each patient spent watching cinematic content. Mean ± SD adherence over 12 weeks of therapy was 78% ± 27% (Fig. 3).

### Patient satisfaction

Patient satisfaction with the therapeutic was assessed at the 12-week visit using the standard Net Promoter Score (NPS) question, a common tool used to gauge customer satisfaction^29^. Parents/guardians of patients were asked how likely they would be to recommend the therapeutic to someone else with “lazy eye”, and asked to respond on a scale of 0 to 10, with 0 being not likely at all and 10 being very likely. Mean ± SD response to the question was 9.5 ± 0.7 (Fig. 3), including 6 of 10 parents/guardians who responded with 10. NPS for the therapeutic, calculated as the percentage of 9 or 10 responses less the percentage of 0 to 6 responses, was +90.

### Safety assessment

Safety was assessed by monitoring for new diplopia, new or worsening heterotropia, worsening visual acuity, or other unanticipated adverse events. Worsening heterotropia was defined as an increase of 10 prism diopters or more of misalignment from baseline, and worsening visual acuity was defined as a loss of 2 or more logMAR lines from baseline in either eye. No cases of the above adverse events or any unanticipated adverse events were reported.

### Personalization

**Figure 4:**
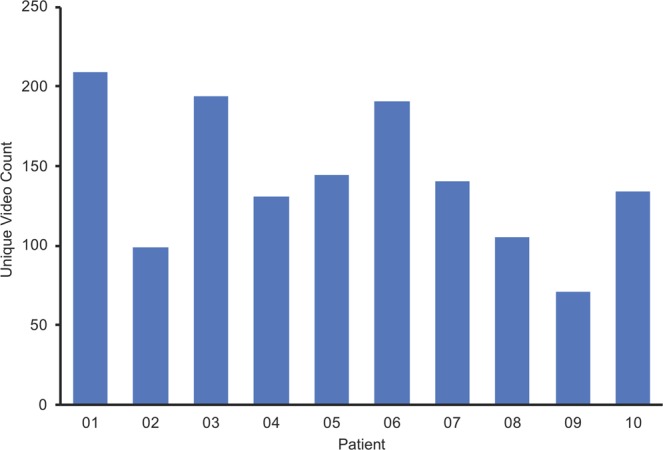
Personalization measurements. Unique video count for each patient (*n* = 10) at study completion.

Content selections of patients were automatically recorded by the therapeutic throughout the study to evaluate the degree to which patients personalized their therapy. A unique video count was calculated for each patient as the number of unique videos selected by the patient over the course of the study. Mean ± SD unique video count was 142 ± 45 (Fig. 4).

## Discussion

In this proof of concept study evaluating the efficacy of the Luminopia One therapeutic, a novel device delivering personalized amblyopia therapy, we report an improvement in mean amblyopic eye visual acuity by 2.9 lines with 12 weeks of therapy. This degree of improvement is greater than those reported in studies on patching and other dichoptic treatments over similar or longer timeframes. Patients of comparable ages improved an average of 2.0 logMAR lines with patching and 1.9 logMAR lines with another dichoptic treatment after 16 weeks of therapy^22^. Using a dichoptic treatment paradigm most similar to our approach, Li *et al*.^23^ reported a mean improvement of 2.0 lines in amblyopic eye visual acuity, though the study included a shorter treatment duration of 2 weeks and therapy was all completed in-office. It is noteworthy that 9/10 patients in our trial had prior amblyopia therapy, which can blunt response to subsequent therapy using traditional modalities^22^.

The high mean adherence of 78% likely contributed to the strong visual acuity improvement. Adherence with the Luminopia One therapeutic was substantially higher than that observed with patching^14,22^. Furthermore, digital technology in the therapeutic monitored adherence objectively to the nearest minute, avoiding the biases of self-reporting^22^ and the imprecision of single daily reports^25^. Compared to studies which also monitored adherence objectively over a similar duration, adherence with the therapeutic was higher and more sustained than other dichoptic therapies^22,26^. The broader library of video content likely ensured that higher adherence could be maintained over a longer treatment period. In addition, the high number of unique videos selected by patients over the course of the study suggests that personalization may have contributed to high adherence through patient engagement.

The lack of adverse binocular events in this study is consistent with the safety profile of other types of amblyopia therapy, including dichoptic treatments^22,23^. The absence of other unanticipated adverse events provides initial support for the overall safety of the therapeutic.

Limitations of this study include the small sample size, lack of comparison group, and lack of long-term outcomes. To further evaluate safety and efficacy of the therapeutic, a future study would ideally be designed as a randomized controlled trial that compares the Luminopia One therapeutic to standard of care treatments such as optical treatment or patching. Future trials should also consider post-treatment follow-up visits to evaluate the long-term outcomes of the therapeutic and retention of treatment benefit. While this study demonstrates initial promise for the positive impact of therapy personalization on adherence and treatment outcomes, future studies can analyze patient behavior in more detail to better understand the relationship.

Importantly, we successfully designed and developed a novel digital therapeutic to test whether an engaging, personalized therapy would improve adherence and treatment outcomes in the home, and our proof-of-concept study provided strong support for the hypothesis in the initial indication of amblyopia. More broadly, the therapeutic provides a successful blueprint for improving treatment outcomes for additional indications in which digital technology, patient engagement, and therapy personalization can improve patient care.

## Methods

### Study design

This study was conducted in the United States in accordance with the principles of the Declaration of Helsinki from the International Conference on Harmonization (ICH). The trial had a single-arm, single center, open-label design and was approved by the Alpha Institutional Review Board. The trial was registered on ClinicalTrials.gov (NCT02782117, Registration Date: May 25, 2016). Parents and/or guardians for all patients provided written informed consent prior to all study-related procedures, and patients aged 7 provided assent to be in the trial as well.

Patients (*n* = 10) participating in the trial were diagnosed with unilateral amblyopia associated with strabismus, anisometropia, or both. Patients were required to be between the ages of 4 and 7, to have sufficient refractive correction at baseline (within 0.75D spherical equivalent of latest cycloplegic refraction) and to have moderate amblyopia, defined as amblyopic eye BCVA between 20/40 and 20/200 at baseline. Patients wore the same refractive correction for the duration of the study. Patients with high myopia greater than −6.00D spherical equivalent (SE) in either eye, previous intraocular or refractive surgery, light-induced epilepsy, or severe developmental delays were excluded. The eligibility criteria were established prospectively.

The primary objective of the trial was to evaluate the efficacy of the Luminopia One therapeutic, and the primary endpoint of the trial was change in amblyopic eye BCVA from baseline to the 12-week visit. The primary objective and endpoint of the trial were established prospectively. After enrollment into the study, patients were provided with a therapeutic and trained on how to select videos and receive treatment. Patients took the therapeutic home and were prescribed treatment for 1 hour per day, 7 days per week for 12 weeks.

### Statistical analysis

Descriptive statistics (mean, standard deviation) and confidence intervals were calculated using Microsoft Excel (version 16.23). Improvements in amblyopic eye BCVA were assessed using a two-tailed, Wilcoxon paired signed rank test using Prism 8.2 (GraphPad Software, San Diego, CA). In all cases, *p* < 0.05 was considered statistically significant.

## Supplementary information

Data File S1.

## Data Availability

All data associated with this study are available in the main text or the supplementary materials.

## References

[1] DiMatteo MR, Haskard KB, Martin LR, Williams SL (2005). The challenge of patient adherence. Ther. Clin. Risk Manag..

[2] Barash CI, Pursel M, Vogenberg FR (2010). Personalized Medicine. P&T..

[3] Schwaederle M (2015). Impact of Precision Medicine in Diverse Cancers: A Meta-Analysis of Phase II Clinical Trials. J. Clin. Oncol..

[4] Pediatric Ophthalmology/Strabismus Preferred Practice Pattern Panel. (2018). Amblyopia Preferred Practice Pattern. Ophthalmol..

[5] Corliss D, Rutstein RP (1999). Relationship between anisometropia, amblyopia, and binocularity. Optom. Vis. Sci..

[6] Ciuffreda KJ, Hokoda SC, Hung GK, Selenow A, Semmlow JL (1983). Static aspects of accommodation in human amblyopia. Am. J. Optom. Physiol. Opt..

[7] Levi DM, McKee SP, Movshon JA (2003). The pattern of visual deficits in amblyopia. J. Vision.

[8] Repka MX (2008). Monocular oral reading performance after amblyopia treatment in children. Am. J. Ophthalmol..

[9] Beauchamp GR, Brown GC, Brown MM, Membreno JH, Sharma S (2002). A cost-utility analysis of therapy for amblyopia. Ophthalmol..

[10] Pediatric Eye Disease Investigator Group. (2008). A randomized trial of atropine versus patching for treatment of moderate amblyopia: follow-up at age 10 years. JAMA Ophthalmol..

[11] Repka MX (2014). Atropine vs patching for treatment of moderate amblyopia: follow-up at 15 years of age of a randomized clinical trial. JAMA Ophthalmol..

[12] Scheiman MM (2008). Patching vs atropine to treat amblyopia in children aged 7 to 12 years: a randomized trial. JAMA Ophthalmol..

[13] Scheiman MM (2005). Randomized trial of treatment of amblyopia in children aged 7 to 17 years. JAMA Ophthalmol..

[14] Wallace MP (2013). Compliance with occlusion therapy for childhood amblyopia. Invest. Ophthalmol. Vis. Sci..

[15] Fielder AR, Moseley MJ, Stephens DA, Stewart CE (2004). Treatment dose-response in amblyopia therapy: the Monitored Occlusion Treatment of Amblyopia Study (MOTAS). Invest. Ophthalmol. Vis. Sci..

[16] Baker DH, Hess RF, Mansouri B, Meese TS (2007). Binocular summation of contrast remains intact in strabismic amblyopia. Invest. Ophthalmol. Vis. Sci..

[17] Mansouri B, Thompson B, Hess RF (2008). Measurement of suprathreshold binocular interactions in amblyopia. Vision Res..

[18] Hess RF, Thompson B (2015). Amblyopia and the binocular approach to its therapy. Vision Res..

[19] Kelly KR (2016). Binocular iPad Game vs Patching for Treatment of Amblyopia in Children: A Randomized Clinical Trial. JAMA Ophthalmol..

[20] Vedamurthy I (2015). A dichoptic custom-made action video game as a treatment for adult amblyopia. Vision Res..

[21] Kelly KR (2018). Improved Binocular Outcomes Following Binocular Treatment for Childhood Amblyopia. Invest. Ophthalmol. Vis. Sci..

[22] Holmes JM (2016). Effect of a Binocular iPad Game vs Part-time Patching in Children Aged 5 to 12 Years with Amblyopia: A Randomized Clinical Trial. JAMA Ophthalmol..

[23] Li SL (2015). Dichoptic movie viewing treats childhood amblyopia. J. AAPOS.

[24] Sauvan L (2019). Contribution of Short-Time Occlusion of the Amblyopic Eye to a Passive Dichoptic Video Treatment for Amblyopia beyond the Critical Period. Neural Plasticity.

[25] Mezad-Koursh D, Newman H, Rosenblatt A, Stolovitch C (2018). Home use of binocular dichoptic video content device for treatment of amblyopia: a pilot study. J. AAPOS.

[26] Bossi M (2017). Binocular Therapy for Childhood Amblyopia Improves Vision Without Breaking Interocular Suppression. Invest. Ophthalmol. Vis. Sci..

[27] Holmes JM (2001). The amblyopia treatment study visual acuity testing protocol. JAMA Ophthalmol..

[28] Beck RW (2003). A computerized method of visual acuity testing: adaptation of the early treatment of diabetic retinopathy study testing protocol. Am. J. Ophthalmol..

[29] Haans H, Raassens N (2017). NPS and Online WOM: Investigating the Relationship Between Customers’ Promoter Scores and eWOM Behavior. J. Serv. Res..

